# A Redução da Frequência Cardíaca após Teste de Esforço é Maior em Adultos Fisicamente Ativos e sem Fator de Risco Familiar para Doença Cardiovascular

**DOI:** 10.36660/abc.20240435

**Published:** 2025-01-22

**Authors:** Larissa de Almeida Dourado, Paulo Magno Martins Dourado, Jaciara Gomes de Oliveira, Evandra Maria da Silva, João Paulo de Almeida Dourado, Pedro Gabriel Senger Braga

**Affiliations:** 1 Clínica Pró-Coração São Paulo SP Brasil Clínica Pró-Coração, São Paulo, SP – Brasil

**Keywords:** Frequência Cardíaca, Doenças do Sistema Nervoso Autônomo, Fatores de Risco, Exercício Físico

## Abstract

O objetivo do presente estudo é investigar a influência da prática de atividade física, na frequência cardíaca (FC) de recuperação após o teste ergométrico, em esteira, em adultos assintomáticos, com e sem fator de risco familiar para doença cardiovascular. Duzentos e cinquenta adultos de ambos os sexos com idades entre 18 e 59 anos foram estudados. Nenhum dos participantes tinham histórico de doença cardiovascular e não utilizavam remédios para doenças crônicas. Todos foram submetidos ao teste de esforço com o protocolo Ellestad. Os valores de delta foram calculados pela subtração da FC de pico pela FC do primeiro, segundo, quarto e sexto minuto de recuperação. O histórico familiar cardiovascular e de atividade física foram documentados. Para a análise estatística foi feito o teste de ANOVA seguido pelas comparações múltiplas de Bonferroni ou Kruskall-Wallis seguido das comparações múltiplas de Dunn. O delta do primeiro, segundo, quarto e sexto minuto da recuperação, foi menor nos indivíduos que não praticavam atividade física e que não tinham fator de risco cardiovascular familiar, em comparação aos fisicamente ativos e sem fator de risco familiar. Os valores de delta não foram diferentes entre os fisicamente ativos com fatores de risco cardiovascular em comparação aos fisicamente inativos com histórico familiar nos momentos estudados. A prática de atividade física, em indivíduos sem fator de risco familiar, pode promover melhor controle autonômico, proporcionando maior capacidade de reduzir a FC após o esforço. Isso não foi observado naqueles com fator de risco familiar, pois a prática de atividade física não influenciou a FC de recuperação.

## Introdução

A frequência cardíaca (FC) de recuperação após o teste ergométrico (TE), é um preditor independente de mortalidade cardiovascular em adultos assintomáticos.^1,2^ A capacidade do sistema nervoso autonômico de controlar a FC imediatamente após o TE, reduzindo mais do que 12 batimentos no primeiro minuto da recuperação, está associada ao melhor prognóstico em comparação aos indivíduos que reduzem valores inferiores a este.^3^ Este efeito é atribuído à redução da atividade nervosa simpática e ao aumento do domínio parassimpático;^3,4^ hábitos relacionados ao estilo de vida, como a prática de atividade física (AF), pode diminuir a atividade muscular nervosa simpática pelo aumento do tônus vagal.^4^ Ademais, o risco de desenvolver doença cardiovascular, está aumentado em indivíduos com o risco familiar, que são aqueles os quais os pais e/ou irmão foram diagnosticados com doença cardiovascular ou tiveram algum evento, como infarto agudo do miocárdio (IAM), parada cardíaca (PC), e acidente vascular encefálico (AVE), antes dos 60 anos.^1,3^ O desfecho cardiovascular naqueles com RF é influenciado pelas comorbidades, como a obesidade e o tabagismo, assim como pela presença de doenças cardiometabólicas.^1^ Portanto, entender se a prática de AF pode influenciar a FC de recuperação em indivíduos sem comorbidades, é fundamental para absorvemos marcadores na prática clínica que são bem estabelecidos na literatura científica e devem ser acompanhados na história clínica do paciente. Isso se faz importante, pois foi observado em homens obesos com RF que a redução da FC após o TE está prejudicada, demonstrando mau prognóstico; no entanto, este prejuízo é predominantemente atribuído ao RF presente nesses indivíduos e não à obesidade.^2^ Nosso estudo visa determinar se a prática de AF, em indivíduos assintomáticos que alcançam as recomendações dos 150 minutos semanais de atividade moderada, pode atenuar a influência do RF na FC de recuperação. Portanto, o objetivo do presente estudo é investigar a influência do histórico da prática de AF na FC de recuperação de adultos assintomático com e sem RF.

## Métodos

Foram estudados 250 adultos de ambos os sexos, com idade entre 18 e 59 anos durante os exames rotineiros da Clínica Pró-Coração, formando, então, uma amostra por conveniência. Inicialmente, os pacientes foram avaliados, junto aos seus prontuários médicos, pelo mesmo médico cardiologista. Os critérios de inclusão foram: idade, entre 18 e 59 anos, ausência de queixas cardiovasculares, como dor na nuca e palpitações, estar apto a realizar um TE no protocolo Ellestad de pelo menos seis minutos, atingindo ao menos 85% da FC predita para a idade (220 – idade = FC predita). O protocolo Ellestad foi escolhido por ser aplicável à população a ser estudada, adultos jovens e de meia-idade assintomáticos do ponto de vista cardiovascular, tornando este o protocolo exclusivo para a participação do estudo e para que não haja interferência de outros protocolos, por gerarem diferentes respostas hemodinâmicas. Além disso, o protocolo escolhido oferece maior intensidade frente aos outros, gerando o estresse cardiovascular necessário para avaliação do sistema cardiovascular no tempo adequado, de 6 a 12 minutos. Apesar deste protocolo ser realizado a todo tempo com 10% de inclinação, podendo gerar fadiga periférica ao invés de central, todos os testes incluídos foram, ao menos, submáximos. Os critérios de exclusão foram: diagnóstico e/ou sintomas de doença cardiovascular, eletrocardiograma ilegível, arritmias pré-teste, PAS ≥ 180 mmHg, PAD ≥ 110 mmHg em repouso, limitações ortopédicas para execução do teste, ingestão de cafeína no dia do teste e uso de fármacos para o controle das crônicas, como a hipertensão, diabetes, dislipidemias, hipotireoidismo e hipertireoidismo. Além do uso de fármacos, indivíduos que reportaram estar sobre algum tratamento hormonal não foram incluídos. Por fim, foram excluídos aqueles indivíduos que não tinham conhecimento sobre as informações referentes ao RF. Cabe ressaltar que não houve controle referente aos hábitos alimentares e à rotina de sono dos participantes.

**Figura 1:**
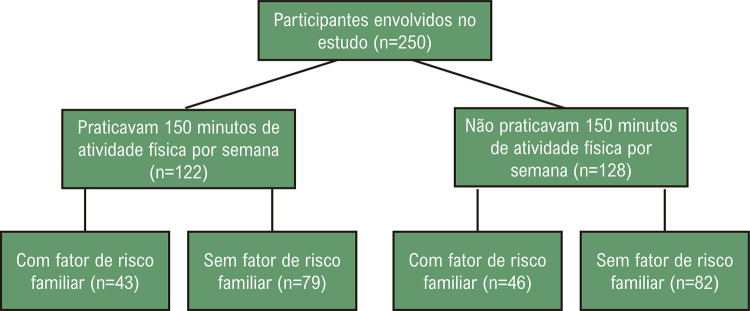
– Fluxo da distribuição dos participantes envolvidos no estudo.

A partir do cálculo amostral de estudos anteriores, utilizamos α=0,05, 1-β=0,80, médias de 16 e 9 e o desvio padrão de 11,2, sendo necessários, ao menos, 41 participantes por grupo.^5^ O estudo foi aprovado pelo comitê de ética e todos os participantes assinaram o termo de consentimento livre e esclarecido. Este estudo está registrado nos Ensaios Clínicos (NCT05987891). As variáveis iniciais, como a pressão arterial (PA) e a FC foram coletadas na esteira, com o participante na posição em pé com os eletrodos no tórax (Inbramed®, Porto Alegre, Brasil). A PA sistólica e diastólica, PAS e PAD respectivamente, foram avaliadas pelo método auscultatório. Todos os aparelhos passam anualmente por manutenções preventivas, garantindo a qualidade do dado analisado. A FC, PAS e PAD foram avaliadas no repouso, no final de cada estágio do teste antes do incremento da esteira, no pico do esforço e no primeiro, segundo, quarto e sexto minuto da recuperação. O pico do exercício foi definido pela incapacidade do participante de continuar realizando esforço naquela carga, referindo exaustão voluntária. Para investigar o controle autonômico, não foram feitas análises da variabilidade da FC e da atividade nervosa simpática. Utilizamos um cálculo que é factível na prática clínica, utilizando o delta da FC de recuperação. Os valores de delta foram calculados pela FC máxima alcançada no teste subtraído pela FC do primeiro, segundo, quarto e sexto minuto da recuperação. Foi cuidadosamente explicado e questionado durante as consultas se os pacientes faziam, ao menos, 150 minutos de AF moderada por semana no último ano. No que tange ao RF, foi perguntado se algum parente, pais e irmãos, foi diagnosticado ou teve algum evento cardiovascular, como o IAM, PC e o AVE antes dos 60 anos. De acordo com os questionamentos, grupos foram formados pela presença (+), ou ausência (-) dos fatores estudados, portanto: não atinge a recomendação de AF e não tem RF (-AF/-RF), atinge a recomendação de AF e não tem RF (+AF/-RF), não atinge a recomendação de AF, mas tem RF (-AF/+RF), e aqueles que atingiam a recomendação de AF e tinha RF (+AF/+RF), conforme demonstrado na Figura 1. Todos os dados foram coletados pelos mesmos autores, que são devidamente treinados e experientes.

### Análise estatística

O teste de normalidade e Kolmogorov-Smirnov foi realizado para verificar a distribuição dos dados. As análises estatísticas foram feitas pelo teste de ANOVA seguido de múltiplas comparações de Bonferroni, quando o dado era normal, ou Kruskal-Wallis seguido pelas comparações múltiplas de Dunn, quando a distribuição do dado não era normal. Foi feita a análise de covariâncias (ANCOVA) seguida de comparações múltiplas de Bonferroni para verificar o impacto do índice de massa corpórea (IMC) e a circunferência abdominal (CA) sobre o delta da FC de recuperação nos grupos estudados. As variáveis categóricas foram analisadas pelo teste de qui-quadrado. Foi considerado estatística se p<0,05. Os dados foram expressos em média ± desvio padrão. Foi utilizado o programa GraphPad Prism, versão 5.0.

## Resultados

Não houve diferença na distribuição de sexo entre os grupos estudados. No que diz respeito ao tabagismo ativo e aos ex-fumantes, a distribuição foi igual entre os grupos estudados: -AF/-RF (n=16), +AF/-RF (n=9), -AF/+RF (n=17) e +AF/+RF (n=8). Na análise de todos os testes, não foram encontradas arritmias, nem testes sugestivos para isquemia do miocárdio em toda a amostra estudada.

A idade foi igual entre os quatro grupos. O IMC e a CA estavam maiores no -AF/-RF do que no +AF/-RF e +AF/+RF (p<0,05; p<0,01, respectivamente), conforme demonstrado na Tabela 1. O tempo de duração do TE foi igual entre os grupos. A FC, PAS e PAD de repouso não diferiram. Entretanto, a FC máxima alcançada durante o teste foi maior no +AF/-RF do que nos -AF/+RF (p<0,05), ainda assim, os valores pressóricos máximos e do período de recuperação foram iguais.

**Tabela 1:** – Variáveis antropométricas e hemodinâmicas dos grupos estudados

Parâmetros/grupos	-AF /-RF (n=82)	+AF/-RF (=79)	-AF/+RF (n=46)	+AF/+RF (n=43)	p
Sexo (F/M)	28/54	34/45	22/24	19/24	0,4258
Idade (anos)	40 ± 10	37 ± 11	42 ± 10	40 ± 12	0,2200
IMC (kg/m^2^)	27,8 ± 3,8 ^a, b^	26,1 ± 4,5	26,9 ± 5,1	25,6 ± 3,7	0,0088
CA (cm)	99,6 ± 11,1 ^c, d^	92,2 ± 13,0	96,9 ± 13,0	91,9 ± 12,2	0,0007
Tempo de exercício (s)	430 ± 102	491 ± 148	454 ± 137	451 ± 117	0,1328
FC (bpm)					
Repouso	81 ± 13	79 ± 14	79 ± 11	75 ± 13	0,1981
Máxima	167 ± 13	171 ± 12 ^e^	164 ± 12	168 ± 13	0,0459
% Predita para a idade	93 ± 6	94 ± 6	92 ± 5	93 ± 5	0,4402
Pressão Arterial Sistólica (mmHg)					
Repouso	121 ± 8	118 ± 10	121 ± 8	119 ± 6	0,1894
Máxima	152 ± 8	150 ± 13	151 ± 9	152 ± 15	0,3373
Pressão Arterial Diastólica (mmHg)					
Repouso	80 ± 5	78 ± 7	79 ± 5	78 ± 6	0,3478
Máxima	80 ± 5	78 ± 7	80 ± 6	78 ± 6	0,3182

**Figura 2:**
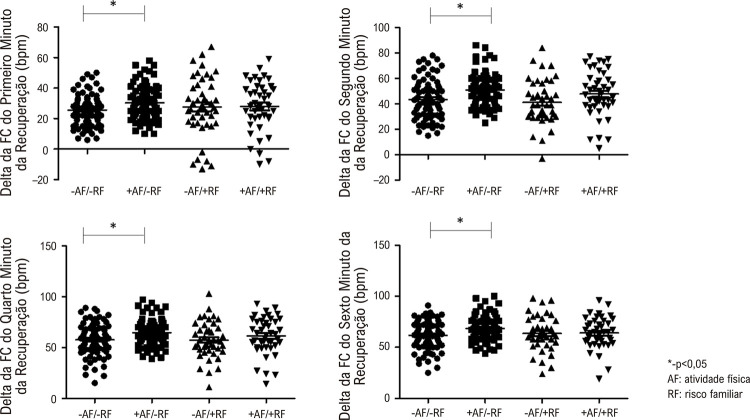
– Valores de delta da FC de recuperação dos grupos estudados.

IMC: índice de massa corporal; CA: circunferência abdominal; bpm: batimentos por minuto; ^a^- -AF/-RF vs. +AF/-RF: p<0,05; ^b^- -AF/-RF vs. +AF/+RF: p<0,05; ^c^- -AF/-RF vs. +AF/-RF: p<0,01; ^d^- -AF/-RF vs. +AF/+RF: p<0,01; ^e^- +AF/-RF vs. -AF/+RF: p<0,05,

Os valores de delta da FC do primeiro, segundo, quarto e sexto minuto foram menores no grupo -AF/-RF em comparação ao +AF/-FR (p<0,05), conforme demonstrado na Figura 2. Não foram encontradas diferenças no delta da FC do primeiro, segundo, quarto e sexto minuto entre os outros grupos estudados. Os valores do terceiro e do quinto minuto não foram documentados pois não são fornecidos automaticamente pelo software utilizado no teste.

A partir das diferenças encontradas do IMC e da CA entre grupos – AF/-RF e +AF/-RF, foi feita a análise para verificar se poderiam influenciar no delta da FC de recuperação como fatores de confusão. Entretanto, apesar do IMC e da CA estarem maiores e os valores de delta menores no grupo -AF/-RF em comparação ao grupo +AF/-RF, mesmo após a análise, a diferença dos valores de delta da FC de recuperação se mantiveram nos grupos estudados (p<0,05), conforme demonstrado na Tabela 2 do Material Suplementar.

## Discussão

Nossos dados mostram a influência do histórico de AF sobre o sistema autonômico, reduzindo com maior efetividade a FC de recuperação em indivíduos sem FR. O grupo -AF/-FR, que demonstrou menor redução na FC de recuperação do que o +AF/-RF, também tinha maior IMC e CA. No entanto, dados mostram que a FC de recuperação é menor nos indivíduos com RF, independente do IMC.^2^ Reforçando os achados do estudo atual em comparação ao supracitado, podemos observar uma possível limitação do histórico de AF em reduzir a FC após o esforço em quem tem RF, uma vez que já foi demonstrado que a prática de AF melhora o controle autonômico em adultos, independente do sexo, idade e IMC.^4^

O papel da prática de AF na melhora do controle da FC, PAS e PAD está bem documentado em pacientes hipertensos não tratados, promovendo maior controle dessas variáveis. Por trás destes efeitos clínicos, é conhecido que o sistema responsável por este efeito, está na redução de disparos da atividade simpática muscular.^6^ Esta, que como uma de nossas limitações, não foi avaliada no presente estudo. A atividade nervosa simpática avaliada de maneira direta, está maior em pacientes sarcopênicos com insuficiência cardíaca, em comparação aos não sarcopênicos. Além disso, os valores de delta da FC do primeiro e do segundo minuto da recuperação após o TE estão menores nesses mesmos pacientes.^7^Esses dados demonstram como a atividade nervosa simpática influencia diretamente a FC de recuperação. Mais adiante, dados similares mostram, em pacientes infartados, que a prática de exercício físico ao longo de seis meses diminui a atividade nervosa simpática e a FC de repouso.^8^

O histórico de RF influencia negativamente o sistema cardiovascular. Quando expostos ao estresse, indivíduos com o RF presente tem a resposta exacerbada e o prejuízo na recuperação da FC.^9^ Inclusive, a presença do RF é mais influente do que a obesidade, no que diz respeito às respostas hemodinâmicas.^2^ Corroborando com estes achados, o presente estudo apresentou que, mesmo após a correção pelo IMC e pela CA. os valores de delta da FC de recuperação se mantiveram, em todos os tempos estudados, entre os grupos -AF/-RF e +AF/-RF. Análises como essa são importantes, pois a documentação do risco familiar por meio de questionário, juntamente a uma medida rotineira da clínica, enriquecem a estratificação de risco. O monitoramento da FC de recuperação é uma medida com uma melhor relação custo-benefício e bem documentada quanto ao seu papel no risco cardiovascular.^1^

O valor de delta da FC de recuperação é um achado que caracteriza a atividade do sistema autonômico sobre a circulação após o exercício físico. Em algumas comorbidades, o desequilíbrio desse sistema é observado, caracterizando a alta atividade simpática e a redução, ou até a inibição, do componente vagal.^10,11^ Inclusive, a hiperatividade simpática retarda a reativação vagal, prejudicando a FC de recuperação. Imediatamente após o exercício, e até o segundo minuto da recuperação, ocorre o predomínio da reativação vagal e a redução da ativação simpática,^11,12^ que pode estar prejudicada na população com RF. É importante ressaltar que a FC de recuperação é influenciada pelas concentrações de norepinefrina, que diminui após o segundo minuto da recuperação,^13^ pela disfunção endotelial,^14^ além de ser dependente de diferentes mecanismos como o comando central, mecanoreflexo, metaborreflexo e a termorregulação.^11^ Esses fatores não foram avaliados no presente estudo.

A ausência da avaliação da atividade nervosa, tal como da variabilidade da FC e registros da AF e do RF mais detalhados são as principais limitações do presente estudo, que, por outro lado, garante futuras investigações sobre a temática dos fatores de risco familiares. Outra limitação envolve a ausência da documentação sobre o sono e alimentação, que podem interferir na FC; entretanto, vale ressaltar que nenhum dos voluntários faziam uso de fármacos de maneira contínua.

## Conclusão

Indivíduos sem RF e que estejam engajados na prática de AF regularmente, têm a maior capacidade de reduzir a FC na fase de recuperação após um TE, em comparação aos não praticantes. Efeitos esses, observados após a correção pelo IMC e CA. Por outro lado, naqueles com RF, a prática de AF não demonstrou influenciar a FC de recuperação. A ausência de avaliações diretas da atividade autonômica é uma limitação deste estudo, assim como uma possibilidade para futuros trabalhos na população com RF.
