# Supplementary material for: A Redução da Frequência Cardíaca após Teste de Esforço é Maior em Adultos Fisicamente Ativos e sem Fator de Risco Familiar para Doença Cardiovascular
Source: Arq Bras Cardiol. 2025 Jan 22;122(1):e20240435. [Article in Portuguese] doi: 10.36660/abc.20240435 (PMC11805530; doi:10.36660/abc.20240435)
Supplement: Translation

# Translation: Heart Rate Reduction after Effort Test Is Higher in Physically Active Adults without Familial Cardiovascular Risk Factor

**DOI:** 10.36660/abc.20240435i

**Authors:** Larissa de Almeida Dourado, Paulo Magno Martins Dourado, Jaciara Gomes de Oliveira, Evandra Maria da Silva, João Paulo de Almeida Dourado, Pedro Gabriel Senger Braga

**Affiliations:** 1 Brazil Clínica Pró-Coração, São Paulo, SP – Brazil

**Keywords:** Heart Rate, autonomic nervous system, Autonomic Nervous System Diseases, Risk Factors, Exercise

## Abstract

The objective of this study is to investigate the influence of physical activity on heart rate (HR) recovery, after treadmill exercise testing, in asymptomatic adults, with and without familial risk factors (FR) for cardiovascular disease. Two hundred and fifty (250) adults of both sexes aged 18 to 59 years were included in the study. None of the participants had a history of cardiovascular disease or used medications for chronic diseases. All individuals underwent exercise testing using the Ellestad protocol. Delta values were calculated by subtracting peak HR from HR in the first, second, fourth, and sixth minutes of recovery. The family history of cardiovascular disease and physical activity were documented. For statistical analysis, ANOVA was performed, followed by Bonferroni or Kruskall-Wallis multiple comparisons, followed by Dunn’s multiple comparisons. The delta values at the first, second, fourth, and sixth minutes of recovery were lower in individuals who did not engage in physical activity and had no family cardiovascular risk factor, compared to those who were physically active and had no family risk factor. No differences in delta values were observed between physically active individuals with cardiovascular risk factors and physically inactive individuals with a family history at the time points studied. In individuals without a family risk factor, physical activity appears to enhance autonomic control, increasing the capacity to reduce HR after exercise. However, this effect was not evident in those with a family risk factor, as physical activity did not impact recovery HR.

## Introduction

Recovery heart rate (HR) after exercise testing (ET) is an independent predictor of cardiovascular mortality in asymptomatic adults.^1,2^ A reduction in HR of more than 12 beats in the first minute of recovery after an ET indicates better autonomic nervous system control. It is associated with a more favorable prognosis, compared to reductions below this threshold.^3^ This benefit is attributed to decreased sympathetic nervous activity and increased parasympathetic dominance;^3,4^ lifestyle factors, such as physical activity (PA), can reduce sympathetic nervous activity in muscles by increasing vagal tone.^4^ Additionally, individuals with a familial risk (FR) of cardiovascular disease – those with parents or siblings diagnosed with cardiovascular disease or who experienced events such as acute myocardial infarction (AMI), cardiac arrest (CA), or stroke (CVA) before age 60 – are at increased risk of developing cardiovascular disease.^1,3^ The cardiovascular outcome in those with FR is influenced by comorbidities, such as obesity and smoking, as well as by the presence of cardiometabolic diseases.^1^ Therefore, understanding whether PA can influence recovery HR in individuals without comorbidities is essential for incorporating well-established markers into clinical practice, which should be routinely monitored in patients’ clinical histories. This is particularly relevant, since in obese men with FR, the reduction in HR following ET was impaired, indicating a poorer prognosis; however, this impairment is primarily attributed to the presence of FR rather than to obesity itself.^2^ Our study aims to determine whether meeting the recommended 150 minutes of moderate PA per week can mitigate the impact of FR on recovery HR in asymptomatic individuals. Thus, the objective of the present study is to examine the influence of PA history, on the recovery HR, of asymptomatic adults with and without FR.

## Methods

Two hundred and fifty (250) adults of both sexes, aged between 18 and 59 years, were assessed during routine examinations at Clínica Pró-Coração, thus forming a convenience sample. Initially, the patients were evaluated, along with their medical records, by the same cardiologist. Inclusion criteria were: age between 18 and 59 years, absence of cardiovascular symptoms (e.g., neck pain, palpitations), ability to complete an ET using the Ellestad protocol for at least six minutes and achieving at least 85% of age-predicted HR (220 - age = predicted HR). The Ellestad protocol was chosen due to its suitability for the young and middle-aged asymptomatic adult population being studied, ensuring a standardized protocol without interference from variations in hemodynamic responses seen in other protocols. Additionally, the chosen protocol provides greater intensity in comparison to the others, thus generating the cardiovascular stress necessary to assess the cardiovascular system within the appropriate timeframe (6 to 12 minutes). While this protocol maintains a 10% incline throughout, which can induce peripheral fatigue rather than central fatigue, all tests were at least submaximal. Exclusion criteria included diagnosed cardiovascular disease or symptoms, unreadable electrocardiogram, pre-test arrhythmias, resting systolic blood pressure (SBP) ≥ 180 mmHg, diastolic blood pressure (DBP) ≥ 110 mmHg, orthopedic limitations, caffeine intake on the test day, or the use of medications for chronic conditions such as hypertension, diabetes, dyslipidemia, hypothyroidism, or hyperthyroidism. In addition to the use of drugs, individuals who reported being on any hormonal treatment were not included. Finally, those not aware of the information regarding the FR were excluded. It is worth noting that there was no control regarding the participants’ eating habits and sleep routines.

Based on the sample size calculation from previous studies, we used α=0.05, 1-β=0.80, means 16 and 9, and a standard deviation of 11.2, requiring at least 41 participants per group.^5^ The ethics committee approved the study, and all participants signed the informed consent form. This study is registered in Clinical Trials (NCT05987891). Initial variables, such as blood pressure (BP) and HR, were collected on a treadmill, with the participant standing with the electrodes on the chest (Inbramed®, Porto Alegre, Brazil). Systolic and diastolic BP (SBP and DBP respectively) were assessed by the auscultatory method. All devices undergo annual preventive maintenance, ensuring the quality of the data analyzed. HR, SBP, and DBP were assessed at rest, at the end of each stage of the test before increasing the treadmill, at peak effort, and in the first, second, fourth, and sixth minutes of recovery. The peak of exercise was defined by the participant’s inability to continue exerting effort at that load, referring to voluntary exhaustion. To investigate autonomic control, analyses of HR variability and sympathetic nervous activity were not performed. A clinically practical calculation was used, based on the delta of recovery HR. Delta values were obtained by subtracting the HR at the first, second, fourth, and sixth minutes of recovery from the maximum HR achieved during the test. During consultations, patients were asked whether they had engaged in at least 150 minutes of moderate PA per week over the past year. For FR assessment, they were asked if any relatives (parents or siblings) had been diagnosed with cardiovascular disease or experienced events like AMI, CA, or stroke before the age of 60. Based on these responses, groups were classified by the presence (+) or absence (-) of PA and FR: those who did not meet the PA recommendation and had no FR (-PA/-FR), those who met the PA recommendation without FR (+PA/-FR), those who did not meet the PA recommendation but had FR (-PA/+FR), and those who met the PA recommendation and had FR (+PA/+FR), as shown in Figure 1. All data were collected by the same authors, who are properly trained and experienced.

**Figure 1:** – Flow chart of the distribution of study participants.

### Statistical analysis

The normality and Kolmogorov-Smirnov tests were performed to verify the distribution of the data. Statistical analyses were performed using the ANOVA test followed by Bonferroni multiple comparisons when the data were normal, or Kruskal-Wallis followed by Dunn’s multiple comparisons when the data distribution was not normal. Analysis of covariance (ANCOVA) followed by Bonferroni multiple comparisons was performed to verify the impact of body mass index (BMI) and waist circumference (WC) on the delta recovery HR in the studied groups. Categorical variables were analyzed using the chi-square test. Statistics were considered if p<0.05. Data were expressed as mean ± standard deviation. GraphPad Prism, version 5.0, was used.

## Results

There was no difference in sex distribution between the studied groups. Regarding active smoking and ex-smokers, the distribution was equal between the studied groups: -PA/-FR (n=16), +PA/-FR (n=9), -PA/+FR (n=17) and +PA/+FR (n=8). In the analysis of all tests, no arrhythmias or tests suggestive of myocardial ischemia were found in the entire sample studied.

Age was the same between the groups. The BMI and WC were higher in the -PA/-FR than in the +PA/-FR and +PA/+FR (p<0.05; p<0.01, respectively), as shown in Table 1. The duration of the ET was the same between the groups. HR, SBP, and DBP at rest did not differ. However, the maximum HR reached during the test was higher in the +PA/-FR than in the -PA/+FR group (p<0.05), with the maximum blood pressure values and recovery period being the same.

**Table 1:** – Anthropometric and hemodynamic variables of the groups studied

Parameters/groups	-PA /-FR (n=82)	+PA/-FR (=79)	-PA/+FR (n=46)	+PA/+FR (n=43)	p
Sex (F/M)	28/54	34/45	22/24	19/24	0.4258
Age (years)	40 ± 10	37 ± 11	42 ± 10	40 ± 12	0.2200
BMI (kg/m^2^)	27.8 ± 3.8 ^a, b^	26.1 ± 4.5	26.9 ± 5.1	25.6 ± 3.7	0.0088
WC (cm)	99.6 ± 11.1 ^c, d^	92.2 ± 13.0	96.9 ± 13.0	91.9 ± 12.2	0.0007
Exercise time (sec)	430 ± 102	491 ± 148	454 ± 137	451 ± 117	0.1328
HR (bpm)					
Resting state	81 ± 13	79 ± 14	79 ± 11	75 ± 13	0.1981
Maximum	167 ± 13	171 ± 12 ^e^	164 ± 12	168 ± 13	0.0459
% Predicted for age	93 ± 6	94 ± 6	92 ± 5	93 ± 5	0.4402
Systolic Blood Pressure (mmHg)					
Resting state	121 ± 8	118 ± 10	121 ± 8	119 ± 6	0.1894
Maximum	152 ± 8	150 ± 13	151 ± 9	152 ± 15	0.3373
Diastolic Blood Pressure (mmHg)					
Resting state	80 ± 5	78 ± 7	79 ± 5	78 ± 6	0.3478
Maximum	80 ± 5	78 ± 7	80 ± 6	78 ± 6	0.3182

BMI: body mass index; WC: waist circumference; bpm: beats per minute; a- -PA/-FR vs. +PA/-FR: p<0.05; b- -PA/-FR vs. +PA/+FR: p<0.05; c- -PA/-FR vs. +PA/-FR: p<0.01; d- -PA/-FR vs. +PA/+FR: p<0.01; e- +PA/-FR vs. -PA/+FR: p<0.05,

The delta HR values of the first, second, fourth and sixth minutes were lower in the -PA/-FR group compared to the +PA/-FR (p<0.05), as shown in Figure 2. No differences were found in the delta HR of the first, second, fourth and sixth minutes between the other groups studied. The values of the third and fifth minutes were not documented since the software does not automatically provide them.

**Figure 2:** – Recovery HR delta values of groups.

Based on the differences found in BMI and WC between the -PA/-FR and +PA/-FR groups, an analysis was performed to verify whether they could influence the delta HR of recovery as confounding factors. Although BMI and WC were higher and delta values were lower in the -PA/-FR group compared to the +PA/-FR group, a significant difference in delta HR values during recovery remained between the groups (p<0.05), as shown in Table 2 of the Supplementary Material.

## Discussion

Our data demonstrate that the history of PA positively influences the autonomic system, reducing recovery HR more effectively in individuals without FR. The -PA/-FR group, which showed a smaller reduction in recovery HR than the +PA/-FR group, also had higher BMI and WC. However, the data indicate that recovery HR is lower in individuals with FR, regardless of BMI.^2^ Supporting our findings, a comparison with previous studies suggests a possible limitation in PA’s effectiveness at reducing HR post-exercise in those with FR, despite evidence that PA improves autonomic control in adults across sex, age, and BMI.^4^

The role of PA in improving HR, SBP and DBP control is well documented in untreated hypertensive patients, promoting greater control of these variables. It is known that the system responsible for these clinical effects is the reduction of muscular nervous sympathetic activity triggers.^6^ This was not evaluated in the present study, comprising one of our limitations. The sympathetic nerve activity assessed directly is greater in sarcopenic patients with heart failure compared to non-sarcopenic patients. Additionally, the delta HR values of the first and second minutes of recovery after ET are lower in these same patients.^7^ These data demonstrate how sympathetic nerve activity directly influences recovery HR. Additionally, other studies in post-infarction patients have shown that six months of physical exercise lowers both sympathetic activity and resting HR.^8^

The history of FR negatively influences the cardiovascular system. When exposed to stress, individuals with FR have an exacerbated response and impaired HR recovery.^9^ In fact, FR is more influential than obesity in terms of hemodynamic responses.^2^ In line with these findings, our study found that even after adjusting for BMI and WC, delta values of recovery HR were consistent at all time points between the -PA/-FR and +PA/-FR groups. Analyses like this are valuable, as documenting family risk via a questionnaire along with routine clinical measurements enhances risk stratification. Monitoring recovery HR is a cost-effective measure whose role in cardiovascular risk is well documented.^1^

The delta value of the recovery HR is a finding that characterizes the activity of the autonomic system on the circulation after exercise. In some comorbidities, an imbalance in this system is observed, characterized by high sympathetic activity and a reduced or inhibited vagal response.^10,11^ In fact, sympathetic hyperactivity delays vagal reactivation, impairing the recovery HR. Following exercise, during the initial two minutes of recovery, vagal reactivation becomes predominant while sympathetic activation decreases;^11,12^ this process may be compromised in individuals with FR. Recovery HR is influenced by norepinephrine levels, which drop after the second minute of recovery,^13^ endothelial dysfunction,^14^ and multiple mechanisms, including central command, mechanoreflex, metaboreflex, and thermoregulation.^11^ These factors were not evaluated in this study.

A limitation of this study is the lack of assessment of nervous activity markers, such as HR variability, and more detailed records of PA and FR, all of which open avenues for future research on family risk factors. Another limitation is the absence of documentation on sleep and diet, which may affect HR; however, it is worth noting that none of the participants were continuous drug users.

## Conclusion

Individuals without FR who regularly engage in PA demonstrate a greater capacity for HR reduction in the recovery phase after an ET compared to non-practitioners, even after adjustments for BMI and WC. On the other hand, in those with FR, PA did not show any influence on recovery HR. The lack of direct autonomic activity assessments remains a limitation, as well as a potential area for future research in populations with FR.
